# Explaining Individual Differences in Motor Behavior by Intrinsic Functional Connectivity and Corticospinal Excitability

**DOI:** 10.3389/fnins.2020.00076

**Published:** 2020-02-05

**Authors:** Jasmine Herszage, Eran Dayan, Haggai Sharon, Nitzan Censor

**Affiliations:** ^1^School of Psychological Sciences – Sagol School of Neuroscience, Tel Aviv University, Tel Aviv, Israel; ^2^Department of Radiology and Biomedical Research Imaging Center, School of Medicine, University of North Carolina at Chapel Hill, Chapel Hill, NC, United States; ^3^Center for Brain Functions, Institute of Pain Medicine, Tel Aviv Sourasky Medical Center, Tel Aviv, Israel; ^4^Sackler Faculty of Medicine, Tel Aviv University, Tel Aviv, Israel

**Keywords:** functional connectivity, motor skill, individual differences, transcranial magnetic stimulation, motor-evoked-potentials, excitability

## Abstract

Motor performance varies substantially between individuals. This variance is rooted in individuals’ innate motor abilities, and should thus have a neural signature underlying these differences in behavior. Could these individual differences be detectable with neural measurements acquired at rest? Here, we tested the hypothesis that motor performance can be predicted by resting motor-system functional connectivity and motor-evoked-potentials (MEPs) induced by non-invasive brain stimulation. Twenty healthy right handed subjects performed structural and resting-state fMRI scans. On a separate day, MEPs were measured using transcranial magnetic stimulation (TMS) over the contrateral primary motor cortex (M1). At the end of the session, participants performed a finger-tapping task using their left non-dominant hand. Resting-state functional connectivity between the contralateral M1 and the supplementary motor area (SMA) predicted motor task performance, indicating that individuals with stronger resting M1-SMA functional connectivity exhibit better motor performance. This prediction was neither improved nor reduced by the addition of corticospinal excitability to the model. These results confirm that motor behavior can be predicted from neural measurements acquired prior to task performance, primarily relying on resting functional connectivity rather than corticospinal excitability. The ability to predict motor performance from resting neural markers, provides an opportunity to identify the extent of successful rehabilitation following neurological damage.

## Introduction

Since most of our daily behavior requires efficient and accurate motor function, studying the motor system has been a central focus of neuroscience research. People vary greatly in their motor performance. Upon initial presentation of a motor task, some will exhibit high motor performance from the first attempt, while others might struggle and get better only through extensive practice. Newly acquired motor skills can be directly evaluated through behavioral performance (for example see Karni et al., 1995; Walker et al., 2003; Korman et al., 2007; Kantak et al., 2010; Censor et al., 2014b; de Beukelaar et al., 2014; Herszage and Censor, 2017; Lugassy et al., 2018), which remains a commonly used and robust measure in studies exploring the motor system. As previously argued (Krakauer et al., 2017), although technological improvements enable an extensive exploration of the nervous system, cognitive neuroscience research is highly dependent on behavioral level measurements. Indeed, behavior undoubtedly detects the quality of skill acquisition and improvements in performance. In motor skill tasks (Karni et al., 1995) for example, better skill acquisition is usually observed as a higher number of accurate sequences tapped in a fixed duration.

In parallel, the motor system is frequently evaluated through multiple modalities and scales, including neural measurements. While these measurements can enrich the collected data beyond behavior *per se*, it might result in even greater variability between measures. Current research lacks the knowledge of a possible integration of data sampled from different measurement levels, limiting the ability to explain individual differences in motor performance.

Could behavior be predicted from an integration of neural measurements acquired prior to task performance? Execution of a motor task requires multiple levels of precise neural processes involving motor planning, motor control and skill acquisition at the central nervous system (Allen et al., 1997; Braitenberg et al., 1997; Muellbacher et al., 2002; Hanakawa et al., 2008; Cohen et al., 2009; Narayana et al., 2014; Gabitov et al., 2016), transformation of the motor command along the corticospinal tract to the peripheral nervous system (Rossini et al., 1994; Di Lazzaro et al., 1998; Groppa et al., 2012), and a correct execution of the movement by the corresponding peripheral muscle.

Correspondingly, motor function can be measured from the central nervous system by assessing functional connectivity within the motor system. This can be achieved through functional MRI scans acquired during resting-state sessions, while measuring the correlation between motor regions of interests (Friston, 1994; Biswal et al., 1995). Such evaluation provides an opportunity to assess the underlying mechanism of motor performance. For example, measurements of functional connectivity before and after motor skill acquisition, showed increased connectivity within the motor system (Taubert et al., 2011). Neuroimaging studies repeatedly show that bilateral primary motor cortex (M1) and the supplementary motor area (SMA) (Perez et al., 2007; Kasess et al., 2008; Dayan and Cohen, 2011) constitute the core motor network not only during active tasks but also during rest, in health and in recovery from stroke (Grefkes et al., 2010; van den Heuvel and Hulshoff Pol, 2010; Kristo et al., 2014).

An additional prominent tool which provides a measurement of the motor system is non-invasive brain stimulation. Transcranial magnetic stimulation (TMS) administered over M1 can induce a movement in subjects’ contralateral hand, known as the motor-evoked-potential (MEP), measured with electromyography (EMG). As such, it reflects the passage of information from the central nervous system toward the peripheral muscle. Single pulse TMS over the M1 in posterior-anterior orientation is known to produce I-waves (Di Lazzaro et al., 1998) which are activated by trans-synaptic corticospinal neurons within M1 (Volz et al., 2014). These signals are thought to reflect the excitability of the underlying motor cortex (Calancie et al., 1999; Hamzei et al., 2006; Di Lazzaro et al., 2008; Hiraoka et al., 2010; Volz et al., 2014; Strigaro et al., 2016), and can be modulated directly via TMS (Volz et al., 2019), or due to motor learning (Tunovic et al., 2014; Ostadan et al., 2016). As such, corticospinal excitability may play an important role in the investigation of the motor system.

While each of the above measurements provides valuable information on the motor system, they are measured at different levels of the motor system, and could thus provide different data sets. A timely goal is to unravel a holistic framework integrating brain and behavior, which could provide the opportunity to unify between multiple levels of analysis. This effort was previously addressed across domains, spanning from molecular to systems neuroscience (Friston, 2010; Love, 2016; Kim et al., 2017). In correspondence with this view, this study aims to integrate three levels of motor system measurements in humans: brain functional connectivity, corticospinal excitability, and behavior. Namely, could subjects’ behavioral performance in a task be predicted from recordings of functional connectivity and corticospinal excitability? Such integration could shed light on individual differences in motor performance, and the neural markers enabling these differences in performance.

## Materials and Methods

### Subjects

A total of 20 healthy volunteers (8 males and 12 females; mean age = 26.1 ± 0.8 years) participated in the study. Subjects were all right handed. Five additional subjects were excluded from the experiment: three subjects were excluded before receiving TMS due to artifacts in the MRI scans, and 2 subjects stopped their participation during the TMS session due to discomfort. All subjects provided written informed consent and all procedures were in accordance with a protocol approved by the Tel-Aviv Sourasky Medical Center and Tel-Aviv University’s Ethics committees. Musicians and video-gamers (past or present) were excluded from the study, as well as subjects with psychiatric or neurological history. In addition, subjects were required to sleep at least 6 hr before each of the experimental sessions.

### Procedure and Task

**FIGURE 1:**
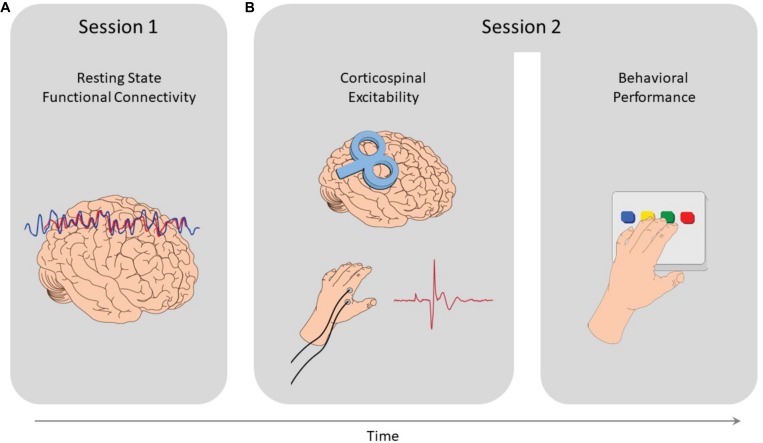
Study design. **(A)** In the first session resting-state functional MRI scans were acquired, from which functional connectivity measures where extracted. **(B)** Then, in the second session, MEPs were induced via single pulse TMS applied over the right M1, and measured from the left FDI muscle to quantify corticospinal excitability. At the end of that session, participants performed a sequential finger tapping task, to measure their motor performance.

The study comprised of 2 sessions (see Figure 1). Subjects first underwent an imaging session where resting-state scans were acquired, in which they were instructed to keep their eyes closed and not fall asleep. Then, on a different session, subjects received single pulse TMS over the right M1 (see details below), while measuring their measuring their MEPs. At the end of this session, following the MEP recordings, subjects performed a sequential finger-tapping task, with their non-dominant left hand. During the task, participants were required to repeatedly tap a 5-element sequence of finger movements (4-1-3-2-4 or 1-4-2-3-1, constantly displayed on the screen), for 30 s, as quickly and accurately as possible. Tapping movements were performed using a 4-key response box (Cedrus Lumina LU440) which was placed in front of the subjects at a comfortable distance and height. Response data were collected for offline analysis using Psychtoolbox (Matlab 8.4). During the task, the sequence was displayed at the middle of the screen and remained in this position throughout the task. The number of correct sequences tapped served as the primary behavioral outcome measure, a common and highly replicable end-point measure for performance in motor sequence tasks (Karni et al., 1995; Walker et al., 2003; Korman et al., 2007; Censor et al., 2010, 2014a,2014b; de Beukelaar et al., 2014). The full procedure included two additional rTMS sessions, conducted after session 2, and thus assured no interfering outcomes. rTMS in those sessions was applied over the lateral prefrontal cortex and vertex, designed to probe the role of human prefrontal cortex in successful reinforced skill formation, reported elsewhere (Dayan et al., 2018).

### Non-invasive Brain Stimulation

Transcranial magnetic stimulation was administered using a Magstim^®^ 70 mm double coil, placed over the right hand-knob area of M1, oriented at 45° to the midsagittal line at a posterior-anterior (PA) direction, with interstimulus intervals jittered between 3–4 s. Individual resting motor thresholds (RMT) were defined as the minimal M1 stimulation intensity yielding five out of ten motor-evoked potentials (MEPs) greater than 0.05 mV in the left first dorsal interosseous (FDI) muscle (Rossini et al., 1994). Brainsight^®^ 2 (Rogue Research, Montreal, QC, Canada)^1^ was used to coregister participants’ head and to mark stimulation sites prior to TMS administration. Four landmarks were used for coregistering the participants’ head to their MRI anatomic scan (nasion, tip of the nose, left and right crus of helix).

### Electromyography (EMG)

Electromyography data were measured from the left first dorsal interosseous (FDI) muscle (Rossini et al., 1994), corresponding to the main behavioral task performed with subjects’ non-dominant left hand. MEP recordings of the FDI muscle are the most common measurement for motor corticospinal excitability, and have been reported in previous studies (for example see Di Lazzaro et al., 1998; Tunovic et al., 2014; Volz et al., 2014, along with most of the TMS studies using the finger tapping task, which measured MEP from the FDI to set the motor threshold such as Perez et al., 2007; Censor et al., 2014b; Narayana et al., 2014). Two 10 mm diameter Ag/AgCl surface electrodes were placed on the left FDI muscle, and one additional ground electrode was placed on subjects’ left ulnar tuberosity. MEP data were amplified using a Digitimer D360 amplifier (Digitimer, Welwyn Garden City, United Kingdom) at a gain of 1000×, band-pass filtered 25 Hz to 1 kHz, and notch filtered at 50 Hz. Data were sampled via a Cambridge Electronic Design (CED; Cambridge, United Kingdom) 1401 A/D converter at a rate of 2 kHz and stored on computer using a commercial data collection software (Signal 6.02, CED).

### Corticospinal Excitability

Motor-evoked-potentials amplitudes were extracted from each response to single-pulse TMS. Individual recruitment-curves were measured based on MEP amplitudes with increasing stimulation intensities by 10% of the RMT. 12 stimulation pulses were given at 100% RMT, and 6 pulses per each condition of 110%, 120%, 130%, 140%, and 150% of RMT (van der Salm et al., 2009), in a non-randomized order (Wassermann et al., 1998). Recruitment curves were then individually extrapolated as the average amplitude at each intensity and excitability was quantified as the slope of the recruitment curve (Wassermann et al., 1998; Ward et al., 2006; Rosenkranz et al., 2007; Orth et al., 2008; Schippling et al., 2009).

### Imaging Data Acquisition

Imaging data were acquired with a 3T SIEMENS MAGNETOM Prisma scanner equipped with a 20-channel head coil at the Wohl Institute for Advanced Imaging, Tel Aviv Sourasky Medical Center. Structural images were acquired with a MPRAGE sequence [repetition time/echo time (TR/TE) = 1860/2.74 ms; flip angle = 8°; field of view (FOV) = 256 mm × 256 mm; slice thickness = 1 mm; 208 axial slices]. Resting-state fMRI images were acquired with a gradient echo-planar imaging (EPI) sequence of functional T2^∗^-weighted images [TR/TE = 2000/35 ms; flip angle = 90°; field of view (FOV) = 384 mm × 384 mm; slice thickness = 4 mm; 34 interleaved axial slices per volume]. The functional scans comprised a total of 240 volumes which lasted 8 minutes. The first 3 volumes were discarded to account of T1-equilibrium effects. Two subjects (of the total 20) were scanned with different functional parameters [TR/TE = 3000/35 ms; flip angle = 90°; field of view (FOV) = 672 mm × 672 mm; slice thickness = 3 mm; 46 interleaved axial slices per volume].

### Imaging Data Analysis

Imaging data analysis was performed with Brain Voyager software (R. Goebel, Brain Innovation, Maastricht, Netherlands). Preprocessing of functional images included realignment and slice-time correction, band-pass filtering (0.01 to 0.1 Hz), segmentation of gray-matter, white matter, and cerebrospinal fluid (CSF) and normalization to the MNI template. The data were additionally spatially smoothed with a Gaussian kernel set at 4 mm full width at half maximum. Signals from the segmented white matter and CSF, and the six motion realignment parameters were regressed out of the signal. Subsequently, reference time courses were extracted from core components of the cortical motor system: the right primary motor cortex (M1) handknob area (each subject’s specific stimulation location, contralateral to the left hand from which behavioral and MEP data was measured, as described above), left primary motor cortex and the SMA, set at MNI (−32, −30, and 51) and (1, −21, and 54) correspondingly (Censor et al., 2014a), each defined as a sphere with radial size of 5 voxels. Correlations between these reference time courses were then calculated for each subject. Only significant correlations were considered for further analysis (*p* < 0.05 resulting in *r* > 0.128 for a sample size of *n* = 237 time points). Accordingly, data from 17/20 subjects in whom there were significant resting-state correlation measurements were included for further behavioral and corticospinal analysis.

### Behavioral Data Analysis

Behavioral data were analyzed with SPSS 25 and Matlab 2017a. Behavioral performance was measured by the number of correct sequences tapped, a highly common measure which accounts for both speed and accuracy (Walker et al., 2003; Korman et al., 2007; Censor et al., 2010, 2014b; de Beukelaar et al., 2014; Herszage and Censor, 2017). To test for the relation between functional connectivity and behavior, as well as other pairwise correlations, we computed Pearson’s coefficient. One subject was excluded from the analysis due to extremely high influence value (Cook’s distance = 7.48, see Cook, 1977), hence all of the analyses in this study were conducted with 16 subjects in total. To test for the relation between all three motor measurements (functional connectivity, corticospinal excitability and behavior), a hierarchical multiple regression was conducted with behavioral performance as the dependent variable (Gelman and Hill, 2006).

## Results

**FIGURE 2:**
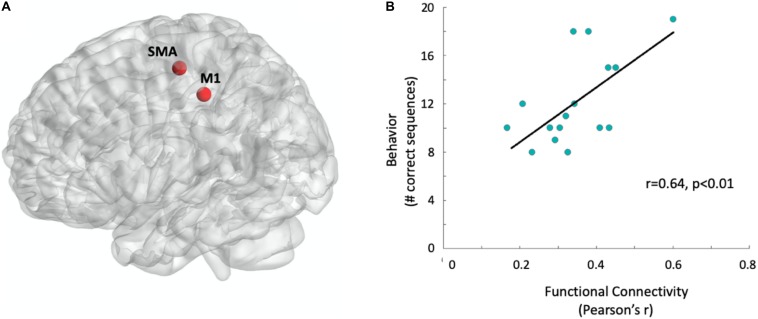
Functional connectivity predicts behavioral performance. **(A)** Sphere regions-of-interest used for the functional connectivity measurement: Supplementary motor area and right M1, contralateral to the left hand which later performed the motor task (see section “Materials and Methods” for MNI coordinates). Regions-of-interest are visualized with the BrainNetViewer (Xia et al., 2013, http://www.nitrc.org/projects/bnv/). **(B)** Correlation between behavior and functional connectivity. Data points were jittered at 2% to minimize overlap. The black line represents the single variant regression line.

To test whether behavior can be predicted from resting-state functional connectivity of the motor system, we first calculated Pearson’s correlation coefficients between the two core regions of the cortical motor system, the contralateral M1 and SMA (Perez et al., 2007; Kasess et al., 2008; van den Heuvel and Hulshoff Pol, 2010; Dayan and Cohen, 2011; Kristo et al., 2014). Indeed, functional connectivity between the contralateral M1 and the SMA (see Figure 2A) significantly correlated with task performance (Pearson’s *r* = 0.64, *p* < 0.01, see Figure 2B) and accounted for *R*^2^ = 40.7% of the variation in participants’ motor behavior. The correlation was not affected by outliers, with Cook’s distance values lower than 0.15, well below the critical threshold of 1.

**FIGURE 3:**
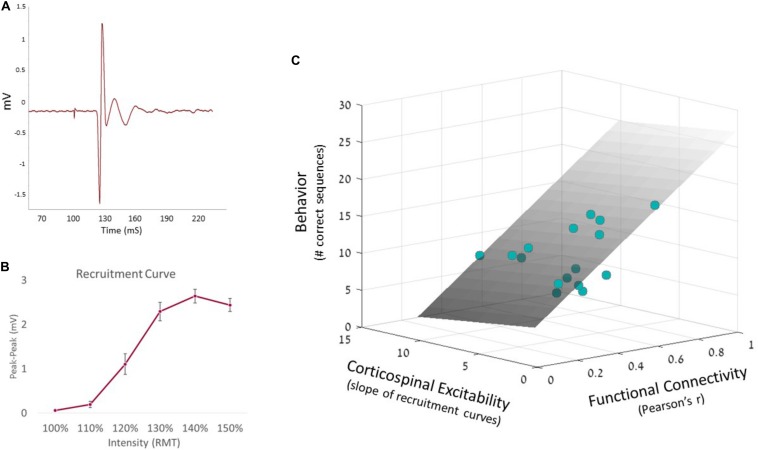
Hierarchical regression model. **(A)** MEP signal, measured from the FDI muscle via EMG system. **(B)** Corticospinal excitability recruitment curves were built based on all individual EMG recordings, averaging MEP amplitudes at each intensity. Error bars represent SEM. **(C)** Predicted behavior is portrayed by the regression plane (gray, see equation above), with the observed single-subject data behavior represented as colored dots. Since the plane is semi-transparent, darker dots represent data points which fall below the plane, while brighter dots represent data points above the plane.

To further examine the predictability of motor behavior, we conducted a hierarchical multiple regression. This enabled to test whether corticospinal excitability could further improve the prediction model. In accordance with the above result, the first step model included functional connectivity which indeed significantly predicted behavior (*F*_1_,_14_ = 9.63, *p* < 0.01). Then, at the second stage of the hierarchical regression, corticospinal excitability was added to the model, resulting in a non-significant contribution (*R*^2^ change = 2.7%, *F*_1_,_13_ = 0.63, *p* = 0.44). Nevertheless, the full model could still significantly predict behavior (ANOVA F_2_,_13_ = 5.0, *p* < 0.03, see Figure 3). The coefficients of the full regression model follow the equation:

Behavior=21.77F*unctionalConnectivity-0.21*E⁢x⁢c⁢i⁢t⁢a⁢b⁢i⁢l⁢i⁢t⁢y+5.31

Prior to conducting the hierarchical multiple regression, the relevant assumptions were tested. Hence, the assumption of singularity was met as the independent variables (corticospinal excitability and functional connectivity) were not significantly correlated (Pearson’s *r* = −0.15, *p* = 0.57). This was further supported by multicollinearity statistics (variance inflation factor = 1.02 for both predictors) which were all within accepted limits, asserting that the assumption of multicollinearity was met (Coakes, 2007). In addition, corticospinal excitability did not correlate directly with behavioral performance (Pearson’s *r* = −0.26, *p* = 0.33), and importantly, an independent two-sample *t*-test comparing behavior with sequence as between group factor showed no difference in performance between sequences [*t*_(__14__)_ = −0.72, *p* = 0.48], confirming an equivalent level of difficulty for both sequences counter-balanced across participants.

Unimanual motor activity is known to activate connections between bilateral M1, known as inter hemispheric inhibition (IHI; for a review see Perez and Cohen, 2009), in addition to frontal areas (such as the SMA). To rule out effects of IHI *per se* (rather than innate differences in unimanual performance), we conducted a control analysis which showed that functional connectivity measured between bilateral primary motor cortices (right and left M1) did not significantly contribute to the model (*R*^2^ change = 1.2%, *F*_1_,_12_ = 0.27, *p* = 0.62), and was not correlated directly with performance (Pearson’s *r* = 0.17, *p* = 0.26).

## Discussion

This study aimed to reveal origins of individual differences in motor skill task performance, by integrating three levels of motor system measurements in humans: brain functional connectivity, corticospinal excitability, and behavior. The results indicate that differences in behavior can be predicted from subjects’ resting-state functional connectivity between the SMA and the contralateral M1, corresponding to the hand performing the task. Specifically, the main finding predicts that individuals with stronger functional connectivity between the contralateral M1 and SMA would exhibit better motor performance. This prediction was not improved nor reduced by corticospinal excitability data, produced from brain stimulation over the same location in contralateral M1.

The main finding of this study, showing that functional connectivity can predict motor performance is in line with previous studies (Mueller et al., 2013; Hamann et al., 2014; Wu et al., 2014). For example, Wu et al. (2014) found that coherence with the region of the left M1 in resting EEG data was associated with motor skill acquisition. In addition, the relation between functional connectivity and behavior was reported in non-motor tasks as well, for example stronger hippocampal connectivity at rest was shown to predict lower episodic memory performance and declining longitudinal memory performance (Salami et al., 2014), while higher hippocampal and posteromedial connectivity at rest predicted better performance in an associative memory task (Wang et al., 2010).

In the current study, resting-state functional connectivity and corticospinal excitability were not significantly correlated. This result is consistent with previous studies investigating the relation between functional connectivity and TMS induced activity (Romei et al., 2007; Fox et al., 2012; Volz et al., 2014; Nettekoven et al., 2015). For example, Volz et al. (2014) reported that functional connectivity did not correlate with MEP latency, another common TMS induced measurement. However, when the TMS coil orientation was changed to an anterior-posterior orientation, different from the orientation used in the current study (see section “Materials and Methods”), Volz et al., found a significant correlation between latency and functional connectivity, indicating that the relation between functional connectivity and TMS induced activity might depend on stimulation parameters.

Corticospinal Excitability did not directly predict behavioral performance in the current study, and indeed, most of the studies linking excitability and behavior focused on time dependent changes in behavior, i.e., learning (for example see Tunovic et al., 2014; Ostadan et al., 2016). Adding to the existing literature, the current results suggest that while corticospinal excitability often changes due to learning processes, it might not be as critical in predicting behavioral performance at early stages of skill acquisition.

Interestingly, even though corticospinal excitability *per se* was not associated with behavior, a combination of functional connectivity and corticospinal excitability was found to predict behavior. Using this unified combination of all three measurements (functional connectivity, corticospinal excitability, and behavior) links data from different levels of measurement into a merged model that can explain the variability in motor performance.

Pairwise relations between these three measurements of the motor system were previously reported to associate with different clinical conditions. For example, the disruption of functional connectivity due to stroke, was found to predict performance impairment (Carter et al., 2010). Furthermore, Grefkes et al. (2010) demonstrated that in patients following subacute stroke, inhibitory TMS over the M1 of the unaffected hemisphere resulted in behavioral improvements and increased connectivity between ipsilesional SMA and M1. In line with these studies, corticospinal excitability was found to be increased in patients with Alzheimer disease (Alagona et al., 2001). Overall, the link of different diseases with functional connectivity and corticospinal excitability has been reported separately in many studies, pointing to potential clinical application using the combination of both measurements not only to predict motor performance in normal populations, but also for the prediction and detection of clinical conditions.

In sum, the current study shows that behavior can be predicted from individuals’ functional connectivity measures, while highlighting the need for additional research into the predictive combination of resting-state functional connectivity and brain stimulation to explain differences in motor performance. Importantly, the ability to predict motor performance from resting state scans in healthy populations supports the utilization of such measurements for clinical use such as assessment of successful rehabilitation likelihood following stroke.

## Data Availability Statement

The datasets generated for this study are available on request to the corresponding author.

## Ethics Statement

The studies involving human participants were reviewed and approved by the Tel-Aviv Sourasky Medical Center and the Tel-Aviv University’s Ethics committees. The patients/participants provided their written informed consent to participate in this study.

## Author Contributions

JH, ED, HS, and NC designed the study and experimental protocol. JH performed the experiments, collected the data, and analyzed the data. JH, ED, and NC wrote and edited the manuscript.

## Conflict of Interest

The authors declare that the research was conducted in the absence of any commercial or financial relationships that could be construed as a potential conflict of interest.

## Footnotes

1 www.rogue-research.com/
